# Stochastic processes drive divergence of bacterial and fungal communities in sympatric wild insect species despite sharing a common diet

**DOI:** 10.1128/msphere.00386-24

**Published:** 2024-08-06

**Authors:** Yu-Xi Zhu, Tian-Yue Yang, Jing-Huan Deng, Yue Yin, Zhang-Rong Song, Yu-Zhou Du

**Affiliations:** 1 Department of Entomology, College of Plant Protection, Yangzhou University, Yangzhou, China; 2 Institute for the Control of the Agrochemicals, Ministry of Agriculture and Rural Affairs, Beijing, China; 3 Entomology and Nematology Department, University of Florida, Gainesville, Florida, USA; University of Wisconsin-Madison, Madison, Wisconsin, USA

**Keywords:** insect, microbial assembly, microbial ecology, source tracking, null model analysis

## Abstract

Arthropods harbor complex microbiota that play a pivotal role in host fitness. While multiple factors, like host species and diet, shape microbiota in arthropods, their impact on community assembly in wild insects remains largely unknown. In this study, we surveyed bacterial and fungal community assembly in nine sympatric wild insect species that share a common citrus fruit diet. Source tracking analysis suggested that these insects acquire some bacteria and fungi from the citrus fruit with varying degrees. Although sharing a common diet led to microbiota convergence, the diversity, composition, and network of both bacterial and fungal communities varied significantly among surveyed insect groups. Null model analysis indicated that stochastic processes, particularly dispersal limitation and drift, are primary drivers of structuring insect bacterial and fungal communities. Importantly, the influence of each community assembly process varied strongly depending on the host species. Thus, we proposed a speculative view that the host specificity of the microbiome and mycobiome assembly is widespread in wild insects despite sharing the same regional species pool. Overall, this research solidifies the importance of host species in shaping microbiomes and mycobiomes, providing novel insights into their assembly mechanisms in wild insects.

**IMPORTANCE:**

Since the microbiome has been shown to impact insect fitness, a mechanistic understanding of community assembly has potentially significant applications but remains largely unexplored. In this paper, we investigate bacterial and fungal community assembly in nine sympatric wild insect species that share a common diet. The main findings indicate that stochastic processes drive the divergence of microbiomes and mycobiomes in nine sympatric wild insect species. These findings offer novel insights into the assembly mechanisms of microbiomes and mycobiomes in wild insects.

## INTRODUCTION

Arthropods are routinely colonized by microorganisms that perform many pivotal functions, ranging from beneficial to deleterious for the host (1–4). Unraveling the mechanisms governing microbiota assembly in arthropods, particularly in wild insects, is considered a major challenge in microbiology (5–7), yet it is essential to predict and manage microbiome dynamics in their applications, such as developing microbial strategies for pest control or protecting the health of economically valuable insects (8–10).

Numerous studies on insect microbiomes have shown that multiple factors, particularly host species and diet, appear to be dominant drivers in shaping microbiota in insects (11–13). There is a hierarchy in the strength of these factors: host species appears to be the strongest, followed by diet (14). For example, Malacrinò (15) analyzed over 4,000 insect samples published in the literature, revealing that host species exert a dominant influence over environmental factors in shaping the diversity and structure of host microbiota. However, comparative studies on beetles, cockroaches, bees, and other species have highlighted diet as a major predictor of microbiota composition (16, 17). Insects can acquire microbes from their diet or surrounding environments, regarded as a regional species pool, leading to rapid and reproducible alterations in their microbiomes (18–21). Nevertheless, despite substantial microbiota variation observed among different insect species or the same insect species with different diets, the underlying microbial community assembly processes as well as the effects of host species or diet on community assembly remain largely unknown.

In a general framework of community assembly, selection, dispersal, and drift are fundamental processes governing microbial communities (5, 22). The niche theory asserts that deterministic processes, such as selection, may drive community convergence in homogeneous environments or divergence in heterogeneous environments (23, 24). In contrast, the neutral theory highlights stochastic processes, including drift and dispersal, in driving the community to diverge or converge (25, 26). Despite deterministic and stochastic forces jointly controlling the host microbial assembly, the relative importance of these processes remains highly controversial (7, 27–29). The magnitude of these processes likely varies among insect species, yet the various geographic ranges, habits, and diets of surveyed insect species might lead to sampling of different environmental pools of microbial species (30). As a result, monocausal effects of host species could seldom be identified in a natural setting, which has hindered the systematic comparison of microbiota assembly across insect species. These limitations can be overcome by analysis of the microbiota in sympatric, ecologically similar species.

In the microhabitat of citrus orchards, we found that multiple insect species with differing taxonomy can share the same diet of citrus. The nine insect species explored in this study exhibit diverse feeding habits: four fly species are polyphagous, two ant species are omnivorous, and two beetles are saprophagous. Despite these differences, all these species feed on citrus. Therefore, these insect species are regarded for sampling a similar environmental pool of microbes. This provided an opportunity to investigate the host specificity of microbial assembly in wild insects, disentangling the effect of environmental factors. In this perspective study, we first investigated the bacterial and fungal communities of nine insect species coexisting sympatrically and sharing a common diet, through applying 16S rRNA and ITS gene high-throughput sequencing. Subsequently, we utilized the microbial source tracking method to investigate the potential origins of microbiota within these insect species. Lastly, we employ the null model analysis to explore the mechanisms of bacterial and fungal community assembly across the nine insect species.

## MATERIAL AND METHODS

### Sample collection

With the specific aim of eliminating the effects of environmental variation on the microbiome of different insect species, we collected six sympatric wild insect species and corresponding citrus fruit tissues from a citrus orchard in Wuxi, Jiangsu, China (31.4201 °N; 120.0908 °E) in September 2023. In brief, the rectangular citrus orchard was divided evenly into six plots, and we randomly selected a citrus tree in the center of each plot. For each tree, all adult insects were collected from the surface or rotting wounds of ripe or abscission citrus fruits by using a sterilized brush or pooter. The corresponding citrus tissues were also collected with a flame‐sterilized spoon. In total, approximately 24 to 60 individuals of each insect species were collected. The individual insect and the corresponding citrus tissues from each tree were considered one replicate, with a total of six replicates per treatment.

All collected insect samples were preserved in 100% ethanol and stored at −20°C until DNA extraction. The frozen insects from each collection were distinguished based on examination of both morphological and molecular characteristics. Nine insect species were obtained: a vinegar fly species (*Drosophila suzukii*] [FDS]), three invasive fruity fly species (*Bactrocera tau* [FBT], *Bactrocera dorsalis* [FBD], and *Bactrocera scutellata* [FBS]), two ant species (*Tetramorium* sp. [ATS] and *Nylanderia* sp. [ANS]), and three beetle species *[Mimemodes monstrosus* (BMM). *Epuraea* (*Haptoncus*) *luteolus* (BEL), and *Phenolia* (*Lasiodites*) *picta* (BPP)]. Before DNA extraction, all the insect individuals were surface-washed with 75% ethanol and then with sterile water three times.

### DNA extraction, library generation, and sequencing

The total DNA of the insect individual and citrus tissues was extracted using a DNeasy Blood and Tissue Kit (Qiagen, Hilden, Germany) and a DNeasy Plant Kit (Qiagen, Hilden, Germany), respectively. We amplified the bacterial 16S rRNA V3–V4 region and the fungal internal transcribed spacer (ITS) ITS1–ITS2 region with the primers 341F (5′‐CCTAYGGGRBGCASCAG‐3′) and 806R (5′‐ GGACTACNNGGGTATCTAAT‐3′) and ITS1F (5′‐CTTGGTCATTTAGAGGAAGTAA‐3’) and ITS2R (5′‐GCTGCGTTCTTCATCGATGC‐3′), respectively, following Zhu et al. (31). PCR was performed using ABI GeneAmp 9700. For both the ITS1/2 amplicon and the 16S rRNA amplicon, three replicates for each sample were amplified using the following protocol: 95°C for 5 minutes, 30 cycles at 95°C for 30 seconds, 52°C for 30 seconds, 72°C for 45 seconds, and a final extension at 72°C for 10 minutes. The purified PCR products were normalized in equimolar amounts before sequencing. The ITS1/2 amplicon library and the 16S rRNA amplicon library were separately sequenced using 250-bp paired-end reads on an Illumina MiSeq 2500 platform by Shanghai Biozeron Co., Ltd. The raw sequence data were filtered for quality control using the DADA2 pipeline. After quality filtering and chimera sequence removal, we obtained a total of 6,507,087 clear reads of bacterial amplicon sequences and 7,426,321 clear reads of fungal amplicon sequences. Bacterial and fungal amplicon sequence variants (ASVs) were classified against the SILVA database and Unite database, respectively. For diversity analysis, all samples were rarefied to the same normalized sequencing depth.

### Microbial diversity analysis

All statistical analyses were conducted in R version 3.6.2 or GraphPad Prism 9.00 (CA, United States). The Shannon diversity index of bacterial and fungal communities was calculated using the vegan package. We used a nonparametric statistical (Kruskal–Wallis test) to test the significant differences in the alpha diversity metric among different host species. The Mann–Whitney test was used to compare the significant differences in Shannon index between fungi and bacteria for all samples. A simple linear regression was performed to survey the correlations between bacterial and fungal diversity indices within all samples. Principal coordinates analysis (PCoA) based on Bray‒Curtis distances was carried out using the pcoa function in the ape package v5.6-2 (32). Permutational multivariate analysis of variance (PERMANOVA) was used to test for significant differences in inoculation treatments using the Adonis function in the vegan package (33).

### Co‐occurrence networks

The network analysis of bacteria, fungi, or bacteria–fungi was constructed using the SpiecEasi package, and only robust correlation with Spearman’s *r* > |0.8| and *P* < 0.05 were plotted using ggClusterNet packages (34). The various topological metrics of each network were evaluated using the vegan and igraph packages.

### Microbial source tracking analysis

The fast expectation‐maximization microbial source tracking (FEAST) method (35) was used to assess the potential origins of bacteria and fungi in each insect species from a potential source diet. The FEAST was carried out with the FEAST package.

### Microbial assembly analysis

Null model analysis was performed to quantify the contribution of ecological processes (i.e., drift, selection, and dispersal) in community assembly based on phylogenetic (β nearest-taxon index [βNTI]) and taxonomic diversity (Raup–Crick index [RCI]) (36). |βNTI| > 2 and |βNTI| < 2 represent, respectively, deterministic processes and stochastic processes that dominate in shaping the community. βNTI < –2 and βNTI > 2, respectively, indicate homogeneous selection and variable selection; |βNTI| < 2 with RCI < –0.95 or >0.95 suggested that the deviation was contributed by homogenizing dispersal or dispersal limitation; |RCI| < 0.95 and |βNTI| < 2 indicates drift in driving the composition of the microbiota. The Mantel test was used to assess the correlation between the βNTI value and host species using the vegan package (31).

## RESULTS

### Bacterial and fungal community variation among different host taxa

**Fig 1:**
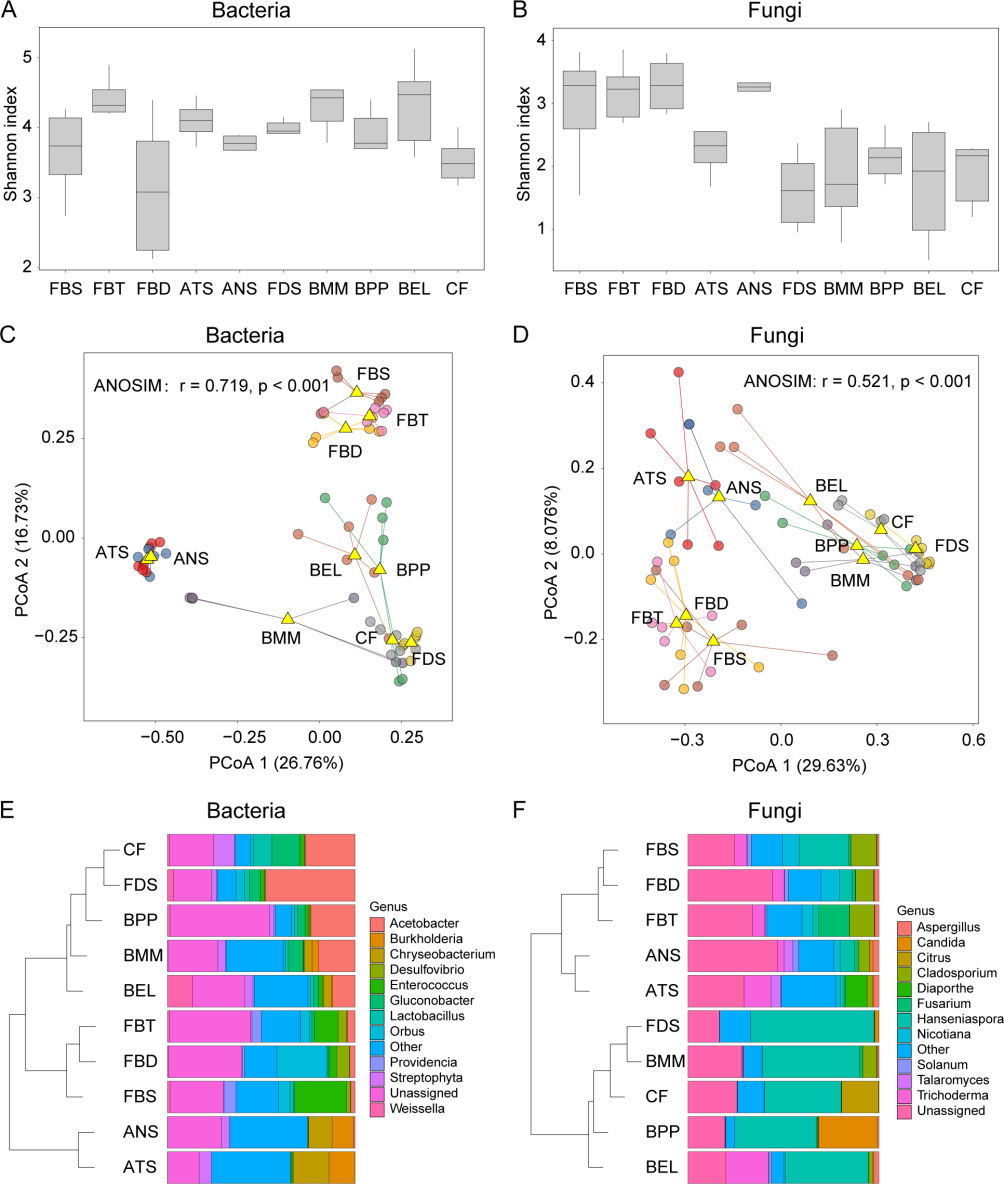
The mycobiome and microbiome of nine insect species and corresponding citrus fruit tissues. Shannon index of the bacterial community (**A**) and fungal community (**B**) in nine insect species and citrus fruit. PCoA of bacterial (**C**) and fungal (**D**) communities in each group. Circles of different colors represent samples from different groups. Composition of bacterial (**E**) and fungal (**F**) communities in different groups. FDS, *Drosophila suzukii*; FBT, *Bactrocera tau*; FBD, *Bactrocera dorsalis*; FBS, *Bactrocera scutellata*; ATS, *Tetramorium* spp.; ANS, *Nylanderia* spp.; BMM, *Mimemodes monstrosus*; BEL, *Epuraea* (*Haptoncus*) *luteolus*; BPP, *Phenolia* (*Lasiodites*) *picta*; CF, citrus fruit. The abbreviations used in the captions of the following figures are identical.

The Shannon diversity indices of both bacterial and fungal communities significantly differed among all samples (Kruskal–Wallis test, bacteria: 18.66, *P* = 0.028, fungal: 28.15, *P* < 0.001) (Fig. 1A and B). Across all insect species, bacterial communities (3.97) exhibited a significantly higher average Shannon index when compared to fungal communities (2.54) (Mann–Whitney test: U = 252, *P* < 0.0001; Fig. S1A). Notably, there was no significant correlation observed between the Shannon index of bacteria and fungi across all insect species (*P* = 0.29; Fig. S1B).

PCoA revealed that both bacterial and fungal community compositions were significantly different among host types, with 43.49% and 37.70% of explaining variation, respectively (ANOSIM, bacteria: *r* = 0.719, *P* < 0.001; fungi, *r* = 0.521, *P* < 0.001) (Fig. 1C and D). Remarkably, in terms of the composition of either bacterial or fungal community, the two ant species (i.e., ATS and ANS) or three tephritidae species (FBT, FBS, and FBD) were clustered separately from other species (Fig. 1C through F). The dominance of certain bacterial taxa such as *Chryseobacterium* and *Burkholderia* in ant species and *Enterococcus*, *Orbus*, *Desulfovibrio*, and *Providencia* in tephritidae species was noted. In addition, *Acetobacter*, *Gluconobacter,* and *Lactobacillus* generally predominated in three beetle species and fly (Fig. 1E). Similarly, specific fungal taxa, including *Talaromyces* in two ant species, and *Cladosporium* and *Nicuotiana* in three tephritidae species exhibited distinct higher abundance (Fig. 1E). The most prevalent fungal genus in three beetle and fly was *Hanseniaspora*, and the relative abundance of the other dominant fungal genera, such as *Candida* and *Trichoderma,* differed among nine insect species (Fig. 1F). Despite the fact that bacterial and fungal composition showed host specificity, the nine insect species exhibited some overlap in both bacteria and fungi taxa (Fig. 1E and F).

### The network of bacteria and fungi in each insect species

**Fig 2:**
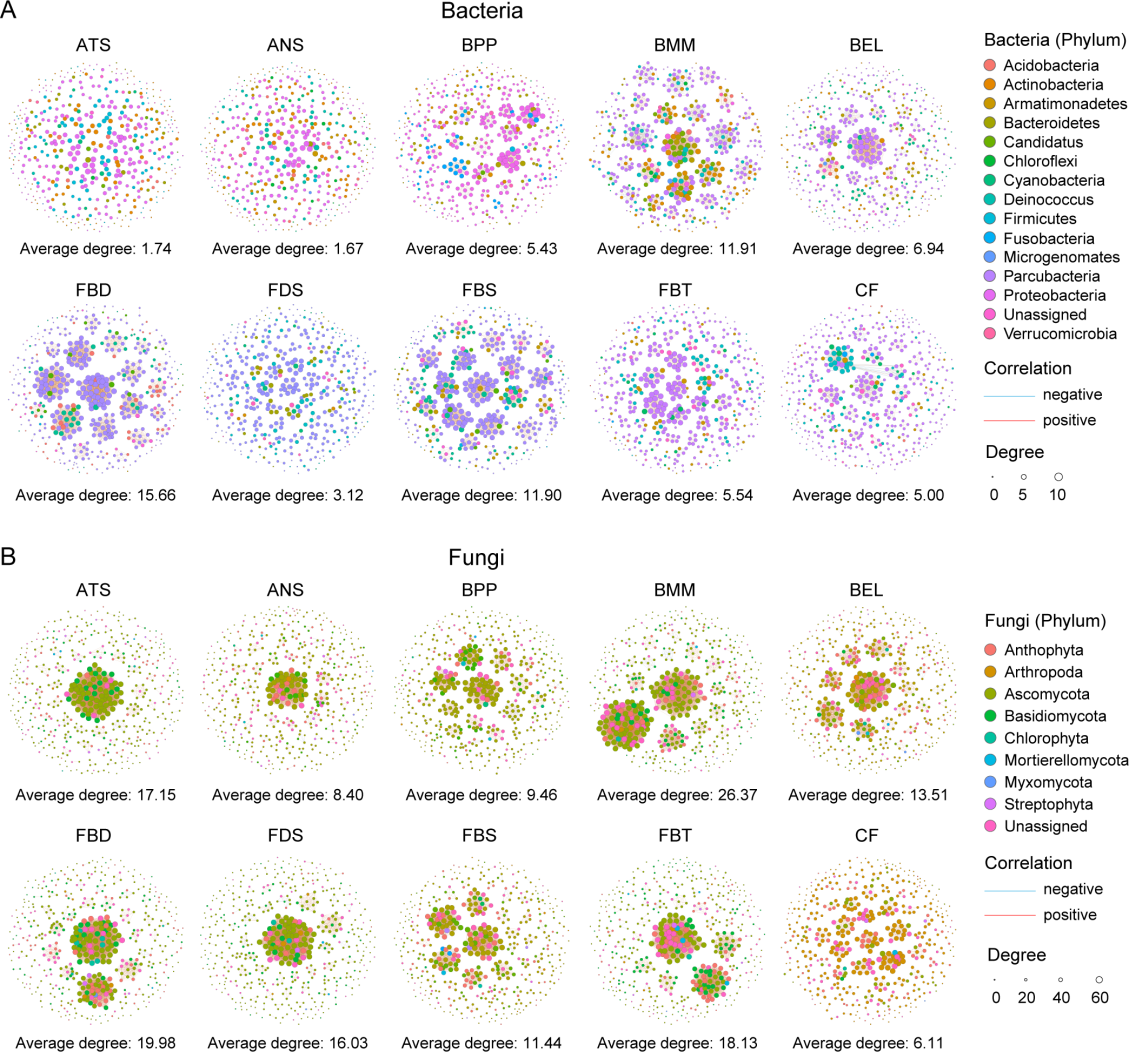
Bacterial (**A**) and fungal (**B**) co-occurrence networks in nine sympatric wild insect species. Edges represent significant Spearman correlations (*r* > |0.8| and *P* < 0.05). Blue and red lines indicate significant negative and positive correlations, respectively. The sizes of the points represent the relative abundances of ASVs in each microbial community.

Patterns of both bacterial and fungal co-occurrence networks differed among the nine insect species (Fig. 2). Bacterial networks exhibited lower complexity, particularly in ant species, while fungal networks displayed higher complexity, with beetle BMM showing the most connections (Fig. 2A and B). The positive relationships dominated in both bacterial or fungal networks across all insect groups, ranging from 61% to 99% for bacteria and 93% to 99% for fungi (Fig. 2; Tables S1 and S2). The taxonomic composition of hub nodes in these networks varied across insect species (Fig. 2).

We also tested the co-occurrence network between bacteria–fungi in each insect species (Fig. S2). Co-occurrence patterns of bacteria–fungi differed among nine insect species, with a higher average degree and more connections in the tephritidae species FBD (4.21, 993) compared to the other species (Fig. S2). The bacteria–fungi network in each insect species was dominated by a positive correlation, implying mutualistic interactions between certain bacteria and fungi in each insect species.

### Microbe source tracking in insects

**Fig 3:**
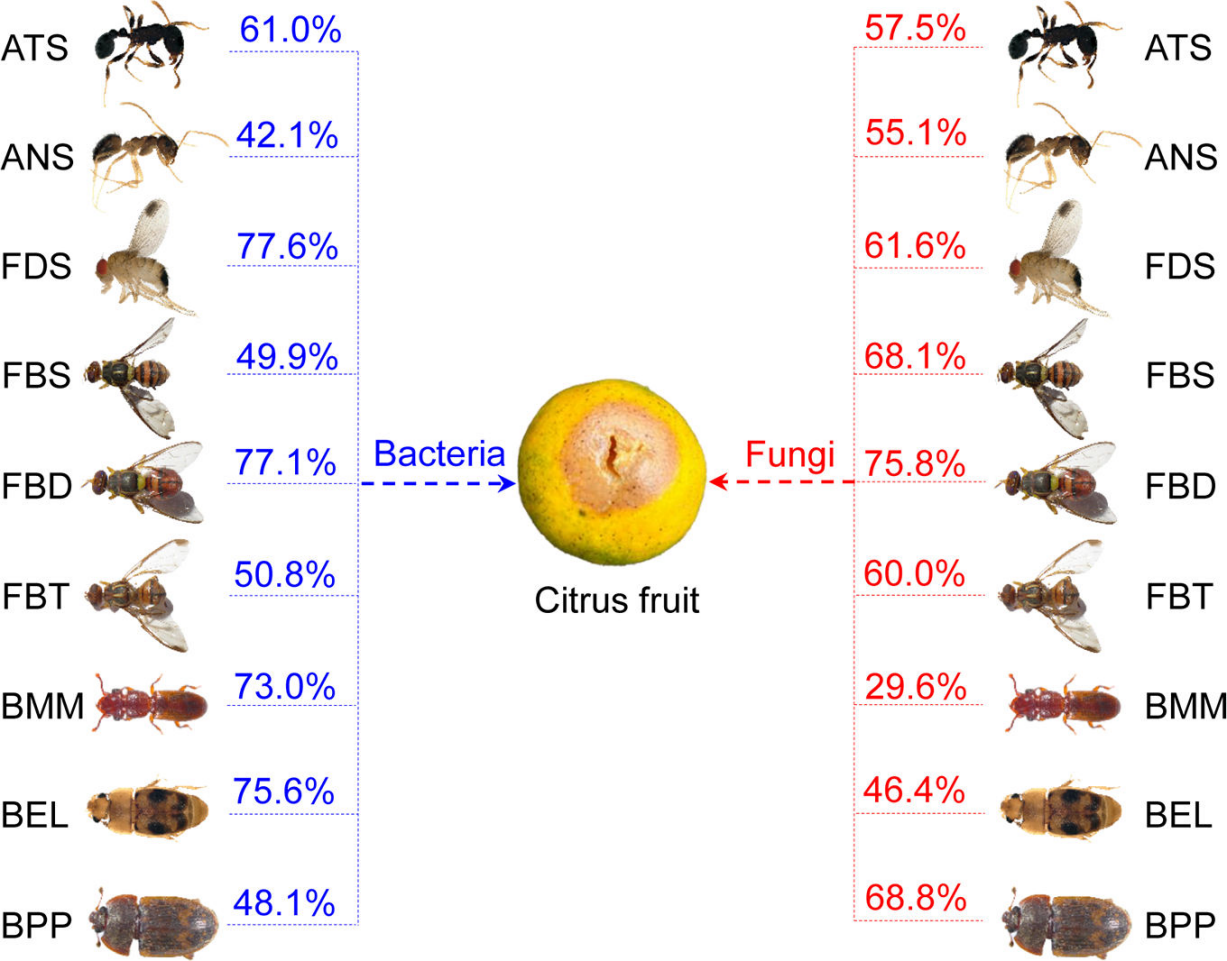
Potential sources of nine insect species-associated bacterial and fungal communities.

Fast expectation‐maximization microbial source tracking (FEAST) revealed that insects acquire about 42.1% to 77.6% of bacteria and 29.6% to 75.8% of fungi from the citrus fruit, and the proportion of obtained microbes was highly correlated with host species (Fig. 3). The flies FDS and FBD obtained the larger majority of bacteria and fungi from the diet compared to other insects (Fig. 3). Two beetles, BMM and BEL, were likely to acquire more bacteria than fungi from their diet (Fig. 3).

### Bacterial and fungal community assembly

Null model analysis showed that both bacterial and fungal community assembly across all insect samples were mainly driven by stochastic processes (−2 < βNTI < 2) (Fig. S3A and C), with dispersal limitation (43.33%) and drift (71.07%) being the prominent factors (Fig. S3B and D). Also, variable selection, a deterministic process, has a substantial contribution in shaping bacterial (44.86%) and fungal (18.03%) community assembly (Fig. S3B and D).

**Fig 4:**
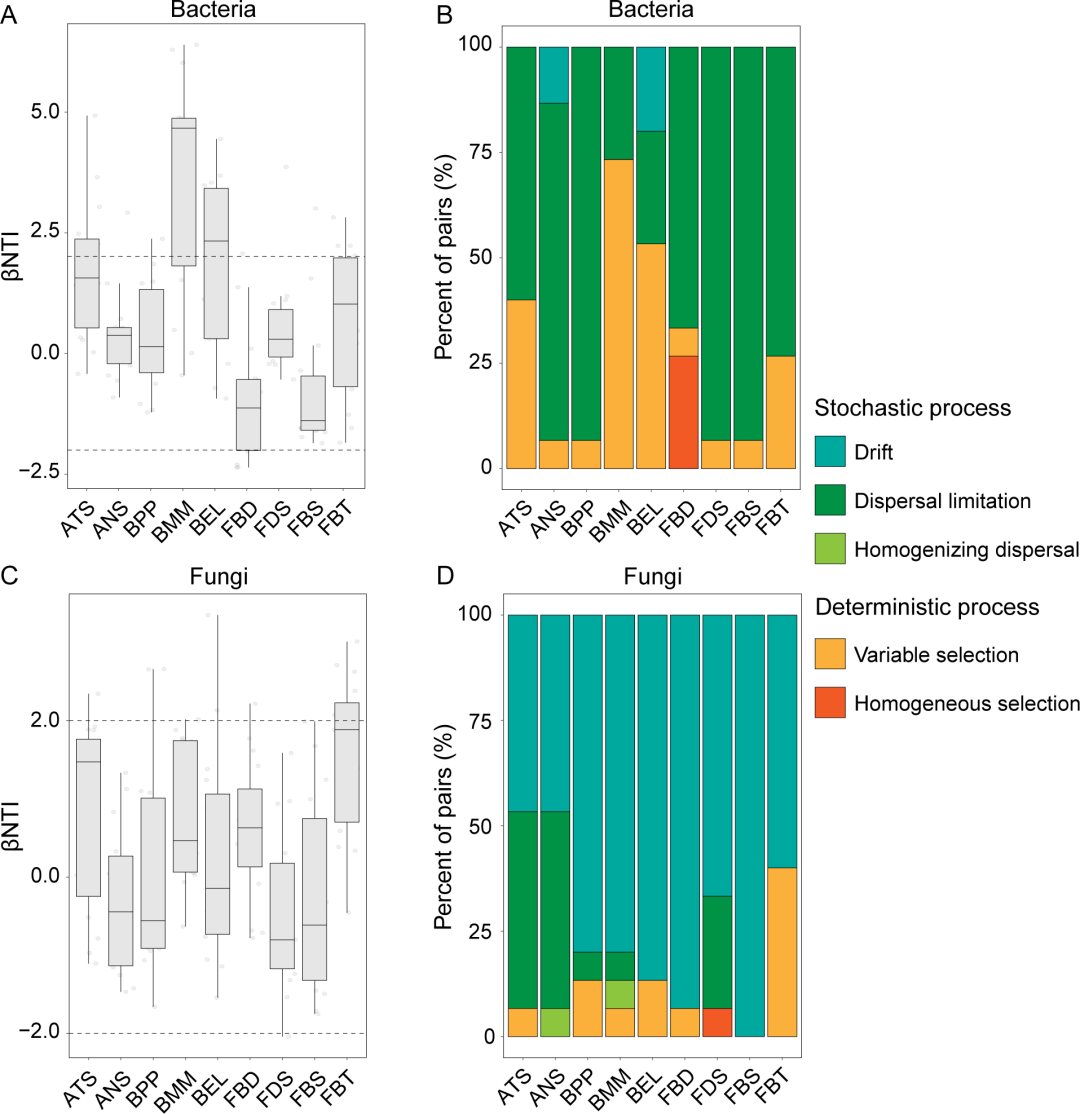
Mechanisms of bacterial and fungal community assembly in nine sympatric wild insect species. The contributions of deterministic (|βNTI| > 2) and stochastic processes (−2 < |βNTI| < 2) on bacterial (**A**) and fungal (**C**) community assemblies in each group. The relative contributions of ecological processes in shaping bacterial (**B**) and fungal (**D**) assemblies in each group.

By analyzing each insect species separately, both bacterial and fungal assemblies from all insect species, except two beetle bacterial communities (BMM and BEL), were dominated by stochastic processes, yet the influence of each process on bacterial and fungal community assembly varied among insect species (Fig. 4). Variable selection was the primary process governing the bacterial communities from two beetle species (BMM: 73.33%, and BEL: 53.33%), while dispersal limitation dominated in driving the bacterial communities from other seven species, with the contribution ranging from 60% to 93% (Fig. 4). In contrast, the process of drift and dispersal limitation dominates in driving the fungal communities from two ant species with approximately equally contribution (46.67%), and drift had a high relative contribution (>60%) to fungal community assembly in each insect species (Fig. 4).

### Host species affecting community assembly

**Fig 5:**
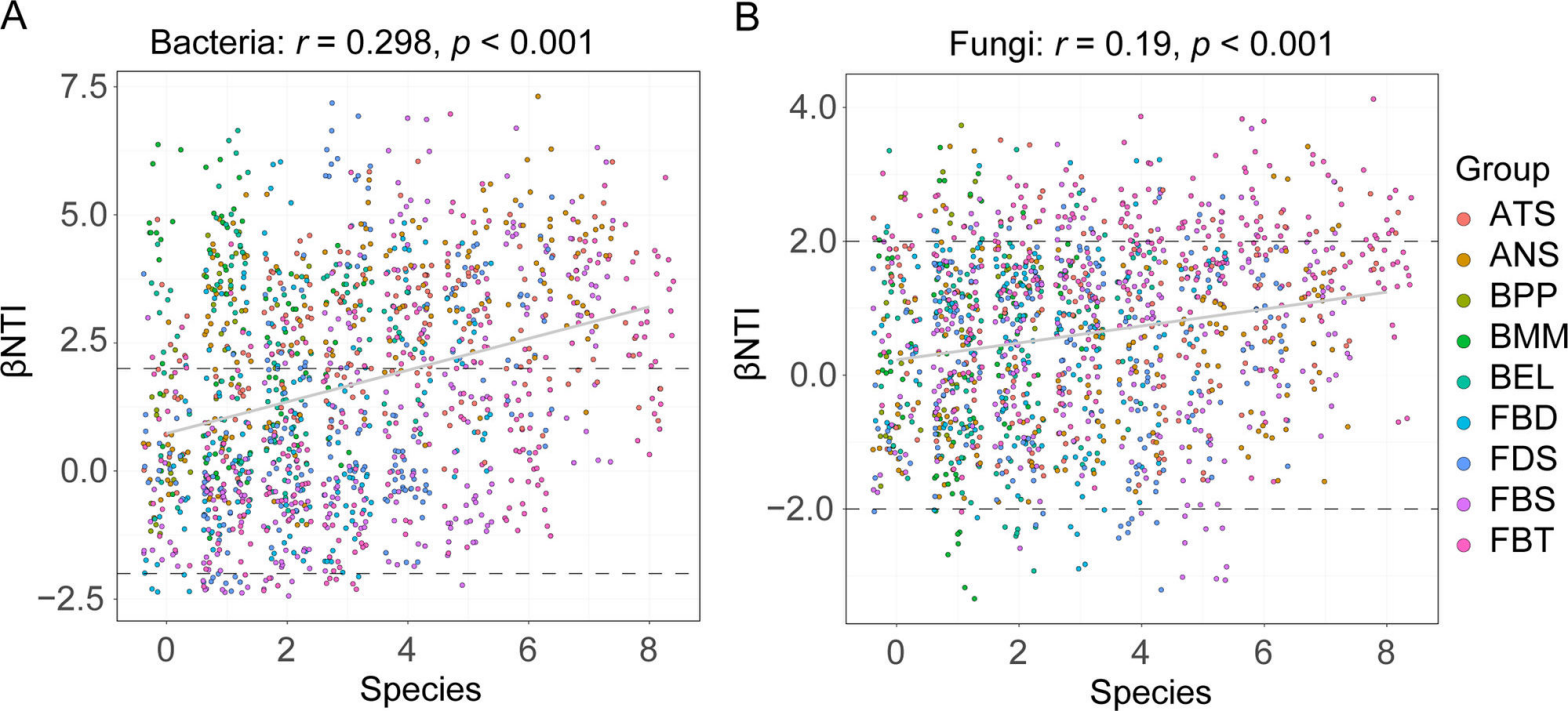
The influence of host species on bacterial (**A**) and fungal (**B**) community assemblies in nine insect species. The *r* and *P*-values were evaluated via the Mantel test.

The Mantel test revealed significant positive correlations between βNTI values of bacterial and fungal community assemblies and insect species, suggesting the significant influence of insect species on the community assembly (bacteria: *r* = 0.298, *P* < 0.001; fungi: *r* = 0.19, *P* < 0.001) (Fig. 5).

## DISCUSSION

Herein, we demonstrate that while sharing a common diet and habitat caused microbiota convergence, the diversity, composition, and network of both bacterial and fungal communities varied significantly across different insect groups. Beyond deterministic processes, such as host morphological and physiochemical differences, and microbe–microbe interaction, stochastic processes emerge as the key drivers in community assembly. The patterns of bacterial and fungal diversity we observed are strongly associated with host-specific community assembly processes.

Our findings agree with the broadly observed pattern that host species shape the bacterial and fungal communities of insects (37–39), resulting in substantial variability in microbiome diversity, composition, and networks across the nine wild insect species we examined. Differences in the community assembly process account for the distinct bacterial and fungal community compositions observed. Strikingly, the variation space of both bacterial and fungal communities was not uniformly distributed; instead, there were three distinct clusters separated by insect groups (Fig. 1C through F). These results indicated that community composition variation is more pronounced among insect groups than within groups, and thus similarity in both microbiome and mycobiome among insect groups likely mirrors the host phylogeny, although evidence of phylosymbiosis requires further exploration (40, 41). While sharing a diet may weaken potential microbiota composition differences among ants, flies, and beetles, each insect species harbors its own specific bacteria or fungi. At the evolutionary and ecological timescales, we speculated host species‐specific microbiota might arise partly through host functional requirements and specific selection during host–microbiota coevolution (31, 42).

Furthermore, we found that sharing a common diet caused some microbiota convergence, and thus showing that diet may play a nonnegligible role in generating bacterial or fungal community succession. Consistent with our findings, previous research has found that the sympatric and diet‐sharing insect species acquire microbes from their diet based on the FEAST source tracking (35), although the magnitude varies between different systems (7, 31), which is likely due to species-specific selection or sampling differences (30). Unlike the previous experiments that focus on gut microbiota without diet contents (31), our comprehensive sampling of complete individuals with diet contents sheds light on the complex interaction between diet, host habitat, and microbial colonization dynamics. Moreover, interspecific interactions within host microbiota, both inherent and introduced, during competition for nutrition and space, can lead to variations in co-occurrence networks (43, 44). Indeed, these microbes directly drawn from related diets or other insects via a hitchhiking effect through the network were impossibly successful in completely colonizing internal and external surfaces due to the host habitat acting as ecological filters (30). Alternatively, diet-mediated insect species-specific microbe exchange across different species also leads to convergence (21). It is noteworthy that interspecific interactions within the host inherent and introduce microbiota during competing for nutrition and space may lead to co-occurrence network variations (45, 46). Overall, while diet has important effects on bacterial and fungal communities, host genetics is still the ultimate force. Considering the universal phenomenon of dietary microbe shifts, it may be an effective approach for pest control by disrupting the intestinal or surface homeostasis through targeted alterations in the diet microbiome.

From an ecological perspective, community assembly processes offer better explanations for microbiome variability across wild insect species. Our findings align with those of previous work demonstrating the dominance of stochastic over deterministic processes in shaping bacterial or fungal community assembly to diverge in honeybees (27), stoneflies (29), and other insects (31). Despite this, the relative contribution of stochastic subprocesses like drift and dispersal differs among insect species. A similar pattern has been described here and previously published, showing the contributions of each assembly process were strongly correlated with host species (27, 29). We thus proposed that the host specificity of the microbiome and mycobiome assemblies was universal in wild insects. Three insect groups, i.e., ants, beetles and flies, have different habitats. Spatiotemporal heterogeneity is also expected to influence microbial succession in these insects. Therefore, despite sharing a common diet, we weaken rather than completely exclude the possibility of the effect of environmental factors on microbial assembly in the sympatric wild insect species (27, 31, 47). Thus, in a natural setting, we argue that host species regulate the trade-off between stochastic and deterministic processes in community assembly, exhibiting active adaptation or passive selection processes under environmental stress.

### Conclusions

In summary, the current study elucidates how different insect species converged to some degree in the microbiome and mycobiome when sharing a common diet or living in sympatry. However, this convergence was insufficient to override the dominant influence of host species. The research can reconcile the importance of both diet and host taxa, while showing that host species are a key driver in shaping insect microbiomes by changing the relative contributions of deterministic and stochastic processes. Our findings contribute to a deeper understanding of the mechanisms underlying microbiome and mycobiome assembly in wild insects.

## Data Availability

Raw sequence data were deposited in the NCBI (accession number PRJNA1100224).

## SUPPLEMENTAL MATERIAL

Supplemental figures and tables msphere.00386-24-s0001.docx Figures S1 to S3; Tables S1 and S2.

ASM does not own the copyrights to Supplemental Material that may be linked to, or accessed through, an article. The authors have granted ASM a non-exclusive, world-wide license to publish the Supplemental Material files. Please contact the corresponding author directly for reuse.
