# Supplementary material for: Stochastic processes drive divergence of bacterial and fungal communities in sympatric wild insect species despite sharing a common diet
Source: mSphere. 2024 Aug 6;9(8):e00386-24. doi: 10.1128/msphere.00386-24 (PMC11351035; doi:10.1128/msphere.00386-24)
Supplement: Supplemental figures and tables — Figures S1 to S3; Tables S1 and S2. [file msphere.00386-24-s0001.docx]

**Supplementary Information for**

**Stochastic processes drive divergence of bacterial and fungal communities in sympatric wild insect species despite sharing a common diet**

Yu-Xi Zhu^1^, Tian-Yue Yang^1^, Jing-Huan Deng^1^, Yue Yin^2^, Zhang-Rong Song^3^, Yu-Zhou Du^1*^

^1^Department of Entomology, College of Plant Protection, Yangzhou University, Yangzhou 225009, China

^2^Institute for the Control of the Agrochemicals, Ministry of Agriculture and Rural Affairs, Beijing 100125, China

^3^Entomology and Nematology Department, University of Florida, Gainesville 32611, Florida, United States.

***CORRESPONDING AUTHORS:**

Yu-Zhou Du, Department of Entomology, College of Plant Protection, Yangzhou University, 88 Daxue South Road, Hanjiang District, Yangzhou City, Jiangsu Province, 225009, China.

E-mail: [yzdu@yzu.edu.cn](mailto:yzdu@yzu.edu.cn)

**This file includes:**

Figure S1 to S3

Table S1 and S2


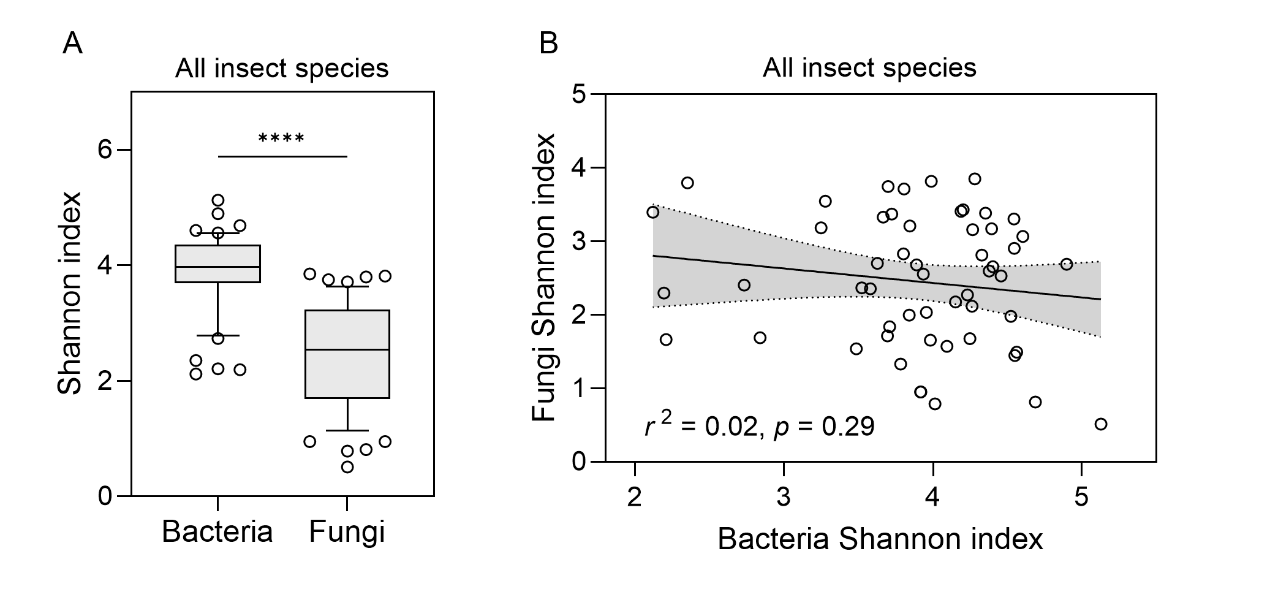


FIGURE S1. (A) Comparison of the Shannon index between bacteria and fungi for all insect samples. (B) The relation between bacteria and fungi alpha diversity index in all insect species.


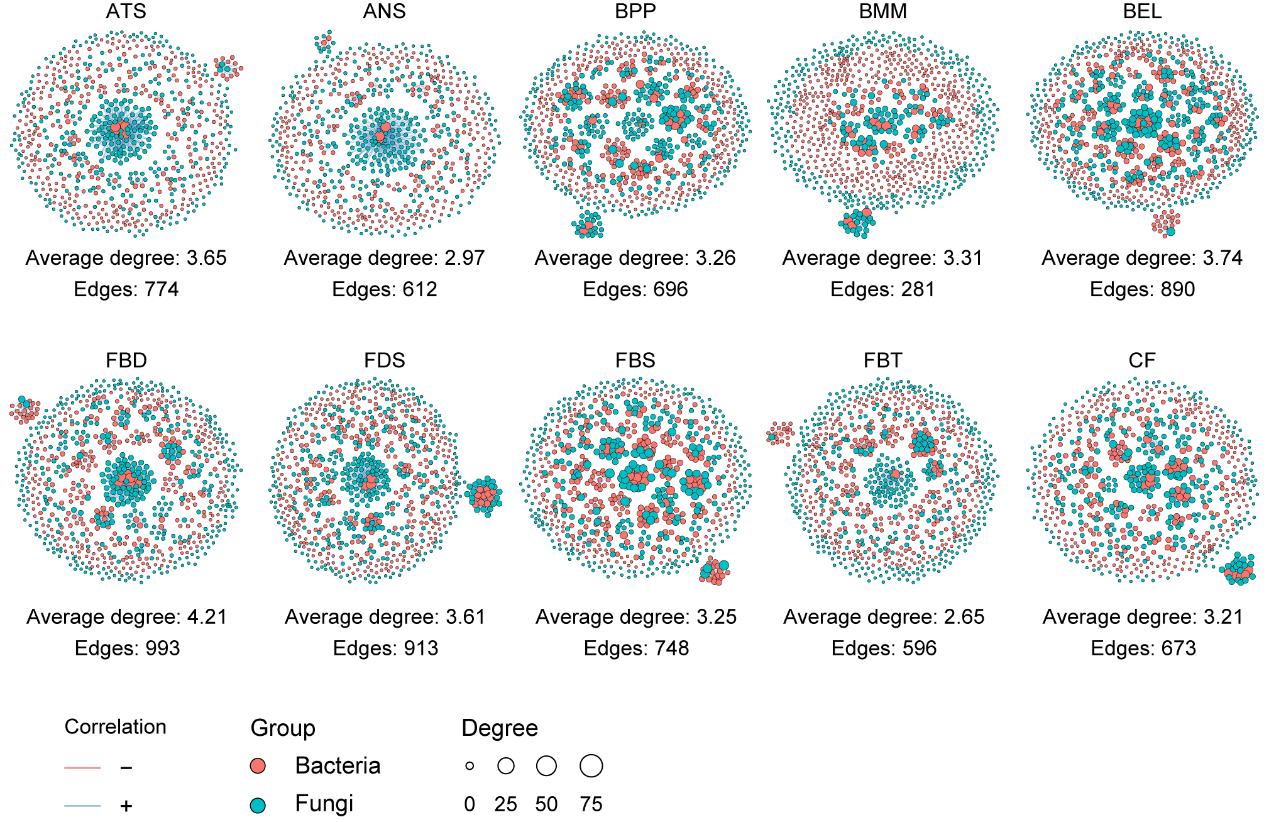


FIGURE S2. Co-occurrence networks of bacteria-fungi in each group. Edges represent statistically significant Spearman correlations (ρ > |0.8|, p < 0.05), whereas blue and red lines, respectively, indicate significant positive and negative correlations.


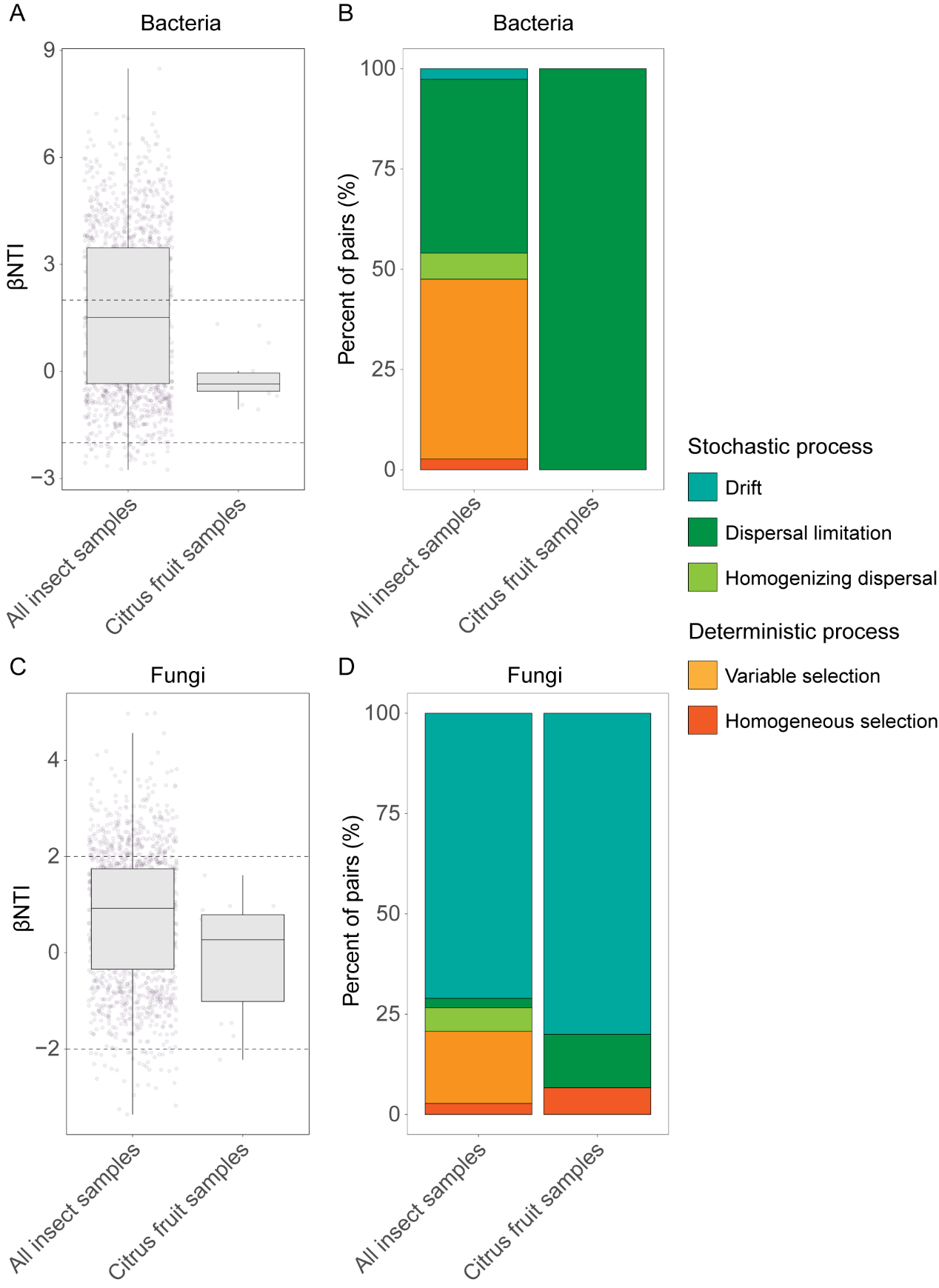
FIGURE S3. Bacterial and fungal community assembly in all insect samples and citrus fruit samples. The contributions of deterministic and stochastic processes on bacterial (A) and fungal (C) community assembly in all insect samples and citrus fruit samples. The relative contributions of ecological processes in shaping bacterial (B) and fungal (D) assembly in all insect samples and citrus fruit samples.

TABLE S1. Bacterial co-occurrence network characteristics in each group.

|  | AB | AR | BB | BG | BL | FB | FF | FJ | FN | O |
| --- | --- | --- | --- | --- | --- | --- | --- | --- | --- | --- |
| num.edges(L) | 273.00 | 272.00 | 1108.00 | 2793.00 | 1382.00 | 3328.00 | 608.00 | 2552.00 | 1182.00 | 1021.00 |
| num.pos.edges | 168.00 | 206.00 | 961.00 | 2384.00 | 1262.00 | 3293.00 | 394.00 | 2501.00 | 1124.00 | 702.00 |
| num.neg.edges | 105.00 | 66.00 | 147.00 | 409.00 | 120.00 | 35.00 | 214.00 | 51.00 | 58.00 | 319.00 |
| num.vertices(n) | 314.00 | 325.00 | 408.00 | 469.00 | 398.00 | 425.00 | 390.00 | 429.00 | 427.00 | 408.00 |
| Connectance(edge_density) | 0.01 | 0.01 | 0.01 | 0.03 | 0.02 | 0.04 | 0.01 | 0.03 | 0.01 | 0.01 |
| average.degree(Average K) | 1.74 | 1.67 | 5.43 | 11.91 | 6.94 | 15.66 | 3.12 | 11.90 | 5.54 | 5.00 |
| average.path.length | 1.00 | 1.00 | 1.38 | 1.63 | 1.24 | 1.06 | 1.18 | 1.17 | 1.04 | 1.86 |
| diameter | 1.00 | 1.00 | 5.91 | 5.91 | 3.94 | 1.97 | 3.94 | 4.93 | 1.97 | 5.91 |
| edge.connectivity | 0.00 | 0.00 | 0.00 | 0.00 | 0.00 | 0.00 | 0.00 | 0.00 | 0.00 | 0.00 |
| mean.clustering.coefficient(Average.CC) | 1.00 | 1.00 | 0.95 | 0.93 | 0.96 | 0.97 | 0.97 | 0.95 | 0.99 | 0.93 |
| no.clusters | 126.00 | 134.00 | 89.00 | 42.00 | 88.00 | 58.00 | 112.00 | 56.00 | 96.00 | 95.00 |
| centralization.degree | 0.01 | 0.01 | 0.03 | 0.05 | 0.08 | 0.06 | 0.02 | 0.03 | 0.02 | 0.05 |
| centralization.betweenness | 0.00 | 0.00 | 0.00 | 0.00 | 0.00 | 0.00 | 0.00 | 0.00 | 0.00 | 0.00 |
| centralization.closeness | 0.00 | 0.00 | 0.22 | 0.43 | 0.19 | 0.09 | 0.11 | 0.20 | 0.04 | 0.35 |
| RM(relative.modularity) | 0.30 | 0.28 | 1.74 | 2.86 | 1.64 | 3.82 | 0.84 | 3.51 | 1.77 | 1.44 |

TABLE S2. Topological property of fungi co-occurrence network in each group.

|  | AB | AR | BB | BG | BL | FB | FF | FJ | FN | O |
| --- | --- | --- | --- | --- | --- | --- | --- | --- | --- | --- |
| num.edges(L) | 3508.00 | 1743.00 | 1925.00 | 5603.00 | 2816.00 | 4066.00 | 3223.00 | 2253.00 | 3454.00 | 1215.00 |
| num.pos.edges | 3450.00 | 1678.00 | 1873.00 | 5548.00 | 2762.00 | 4019.00 | 3157.00 | 2214.00 | 3403.00 | 1168.00 |
| num.neg.edges | 58.00 | 65.00 | 52.00 | 55.00 | 54.00 | 47.00 | 66.00 | 39.00 | 51.00 | 47.00 |
| num.vertices(n) | 409.00 | 415.00 | 407.00 | 425.00 | 417.00 | 407.00 | 402.00 | 394.00 | 381.00 | 398.00 |
| Connectance(edge_density) | 0.04 | 0.02 | 0.02 | 0.06 | 0.03 | 0.05 | 0.04 | 0.03 | 0.05 | 0.02 |
| average.degree(Average K) | 17.15 | 8.40 | 9.46 | 26.37 | 13.51 | 19.98 | 16.03 | 11.44 | 18.13 | 6.11 |
| average.path.length | 1.01 | 1.08 | 1.11 | 1.01 | 1.03 | 1.01 | 1.01 | 1.02 | 1.01 | 1.01 |
| diameter | 2.93 | 4.93 | 3.94 | 3.91 | 4.93 | 2.94 | 2.94 | 4.93 | 2.94 | 2.96 |
| edge.connectivity | 0.00 | 0.00 | 0.00 | 0.00 | 0.00 | 0.00 | 0.00 | 0.00 | 0.00 | 0.00 |
| mean.clustering.coefficient(Average.CC) | 0.98 | 0.97 | 0.95 | 0.98 | 0.96 | 0.97 | 0.97 | 0.97 | 0.97 | 0.98 |
| no.clusters | 98.00 | 109.00 | 86.00 | 64.00 | 78.00 | 82.00 | 97.00 | 83.00 | 81.00 | 91.00 |
| centralization.degree | 0.15 | 0.10 | 0.06 | 0.11 | 0.09 | 0.12 | 0.14 | 0.06 | 0.11 | 0.03 |
| centralization.betweenness | 0.00 | 0.00 | 0.00 | 0.00 | 0.00 | 0.00 | 0.00 | 0.00 | 0.00 | 0.00 |
| centralization.closeness | 0.04 | 0.09 | 0.12 | 0.04 | 0.09 | 0.05 | 0.06 | 0.05 | 0.05 | 0.04 |
| RM(relative.modularity) | 0.46 | 1.06 | 2.32 | 3.88 | 2.66 | 2.89 | 0.83 | 2.64 | 2.62 | 1.78 |
